# An update on multimodal management of craniopharyngioma in children

**DOI:** 10.3389/fonc.2023.1149428

**Published:** 2023-05-05

**Authors:** Laura-Nanna Lohkamp, Ekkehard Matthias Kasper, Alexandra Espinosa Pousa, Ute Katharina Bartels

**Affiliations:** ^1^ Division of Neurosurgery, Department of Surgery, The Hospital for Sick Children, Toronto, ON, Canada; ^2^ Division of Neurosurgery, St. Elizabeth’s Medical Center, Boston University Medical School, Brighton, MA, United States; ^3^ Hamilton Health Sciences, Hamilton, ON, Canada; ^4^ Division of Haematology/Oncology, Department of Paediatrics, The Hospital for Sick Children, Toronto, ON, Canada

**Keywords:** pediatric, craniopharyngioma (CP), diagnosis, multimodal management, function preservation, update

## Abstract

Craniopharyngioma (CP) represent 1.2-4.6% of all intracranial tumors in children and carry a significant morbidity due to their lesional intimacy with structures involved in neurological, visual, and endocrinological functions. Variable treatment modalities being available, including surgery, radiation therapy, alternative surgeries, and intracystic therapies or combinations of them, their common goal is to reduce immediate and long-term morbidity while preserving these functions. Multiple attempts have been made to re-evaluate surgical and irradiation strategies in order to optimize their complication and morbidity profile. However, despite significant advances in “function sparing” approaches, such as limited surgery and improved technologies of radiation therapies, achieving interdisciplinary consensus on the optimal treatment algorithm remains a challenge. Furthermore, there remains a significant span of improvement given the number of specialties involved as well as the complex and chronic nature of CP disease. This perspective article aims to summarize recent changes and knowledge gains in the field of pediatric CP, outlining updated treatment recommendations, a concept of integrative interdisciplinary care and the implication of novel potential diagnostic tools. A comprehensive update on the multimodal treatment of pediatric CP is presented, focusing on “function-preserving” therapies and their implications.

## Introduction

Craniopharyngioma (CP) are the most common non-​neuroepithelial intracerebral neoplasm in children, accounting for 1.2-4.6% of all pediatric intracranial tumors (1, 2). CP can be divided into 2 distinct histomorphological subtypes: the papillary CP (PCP) and adamantinomatous CP (ACP). Latter one accounts for the majority of all pediatric CP and is characterized by the presence of cystic formations, calcifications and molecular genetically by CTNNB1 mutations (1). Histopathologically being benign tumors (WHO grade I), overall survival rates of CP described in children range from 83% to 96% at 5 years (3, 4), and 65% – 100% at 10 years (5–9), averaging 62% at 20 years (10) and are associated with tumor- and/or treatment-related risk factors, such as recurrent or progressive disease, permanent neuroendocrine deficiencies, and cerebrovascular impairment along with impacted quality of life (2, 11). Especially hypothalamic involvement/damage remains one of the most important outcome factors in children with CP (12–14). Therefore, the major goal of treatment is to reduce immediate and long-term morbidity while preserving neuroendocrine and neurological function (15, 16). Variable treatment options are available, including surgery, radiation, intracystic therapies for cystic craniopharyngioma, and/or combinations of them, all intending to control the tumor and its space-occupying effect causing impairment of functional structures. Until recently the treatment of choice in case of favorable tumor location (without hypothalamic involvement) was complete resection (17). However, the morbidity related to radical surgery and radiation, intensified the controversies regarding their role and benefit, stipulating the need for alternative, predominantly “function sparing” therapies (18–21). One concept was to reduce the invasiveness of surgery and to perform “limited surgeries”, including partial resection, cyst fenestration or aspiration, catheter and Ommaya reservoir placement, or cerebrospinal fluid (CSF) diversion (16), either alone or in combination with other treatment modalities (13, 22–26). Furthermore, advances were made in radiation therapy, including technology improvements for toxicity reduction, as well as application of alternative radiation techniques (27–30). The complexity and chronic nature of CP disease has established interdisciplinary management as standard of care. However, achieving interdisciplinary consensus on the most “function sparing” and optimal treatment approach often remains a challenge. This perspective article aims to summarize recent changes and knowledge gains in the field of pediatric CP, including a state-of-the-art diagnostic algorithm and the implication of novel potential diagnostic tools. Different treatments options and concepts of “function sparing” treatments are outlined with a focus on integrative interdisciplinary care.

### Diagnosis, imaging classification and prognostic stratification

The diagnosis of CP in children often gets delayed for various reasons (31, 32). Most of the time children do not appreciate symptoms themselves and therefore depend on care givers or pediatricians to be diagnosed; or symptoms may be very subtle, that they won’t be recognized at all. Furthermore, the clinical picture of CP can be highly variable depending on the tumor location, however is mostly characterized by symptoms of increased intracranial pressure (nausea, headache) (8, 33), visual impairment (62-84%), and endocrine deficits (52-87%) (16). Latter ones are frequently the primary clinical manifestation of tumor-related involvement or proximity to the hypothalamic-pituitary axis and may be often misinterpreted or not attributed to the potential diagnosis of CP. At the time point of diagnosis 40-80% of patients present with at least one endocrine deficit (12, 34, 35). For instance hormonal symptoms such as neurohormonal diabetes insipidus are observed preoperatively in 17–27% of all CP patients (35). Other indicators of endocrine dysfunction may be reduced growth velocity, weight gain, predictive of hypothalamic obesity, and precocious or delayed puberty (31, 36), primarily becoming noticed during routine check-ups. Once the probability of CP diagnosis is given, neuroimaging *via* MRI should be completed. Its importance is reflected in answering the following questions:

1. Do morphology and imaging characteristics (location, cyst, calcification, contrast-enhancement, etc) of the tumor potentially correspond to the diagnosis of CP?
2. Does the tumor have a cystic component and if so, how prominent is it?
3. What are the tumor’s features with respect to location, invasiveness, and extent of neurohypophyseal and hypothalamic damage (grading)

**Figure 1:**
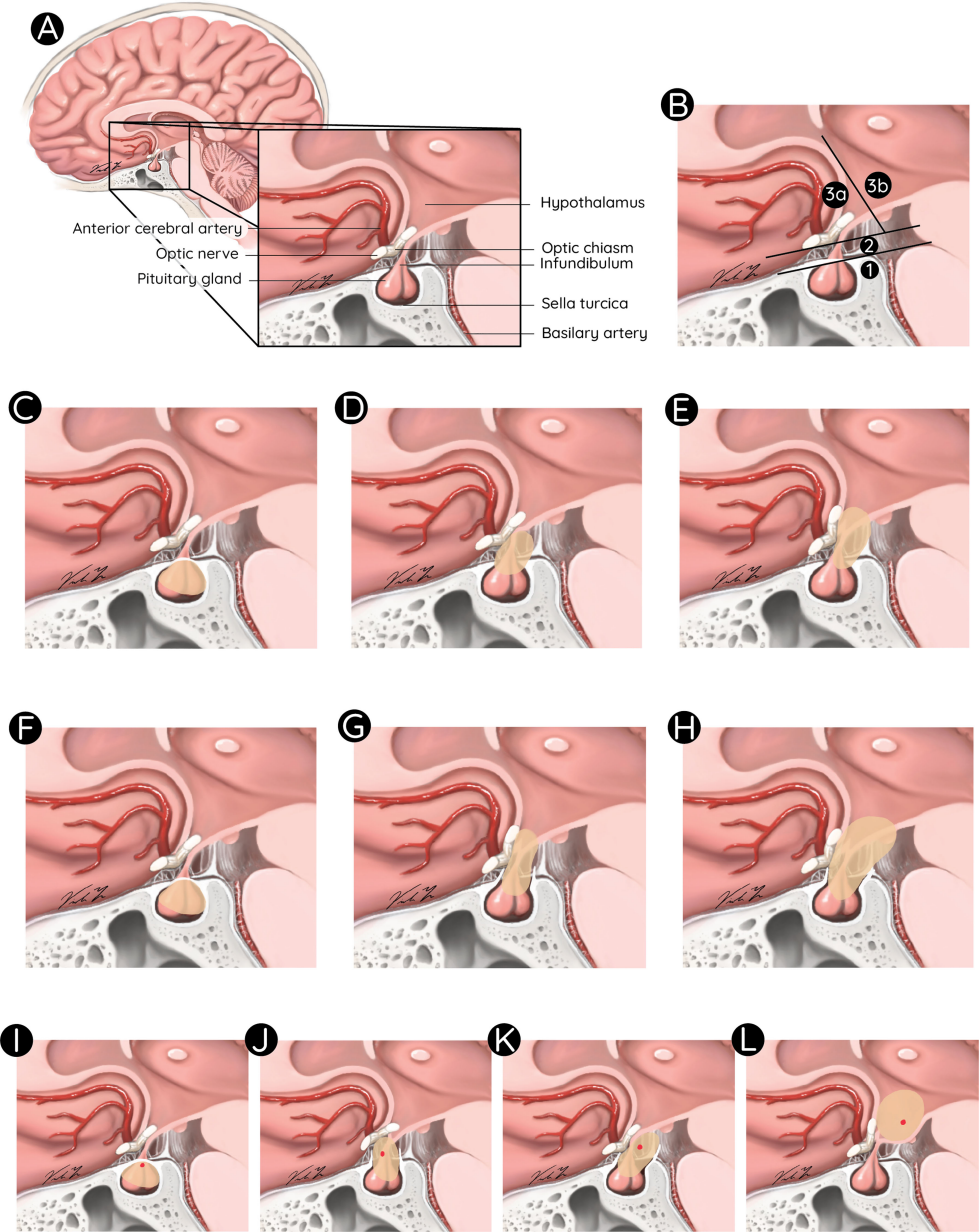
Anatomical classifications for paediatric CP. Anatomical overview of the sella region **(A)**. Classification of pediatric CP according to Flitsch et al.: Type 1, below the diaphragm sellae **(B1)**; Type 2, supradiaphragmatic and infrachiasmatic **(B2)**; Type 3, above the chiasm in front of **(B3a)** or extending beyond the mammillary bodies **(B3b)**, Puget et al.: Grade 0, no hypothalamic involvement **(C)**; Grade 1, tumor displacing the hypothalamus **(D)**; and Grade 2, hypothalamic involvement **(E)**, Müller et al.: **(F–H)**, and Morisako et al.: Type 1, intrasellar **(I)**; Type 2, prechiasmatic **(J)**; Type 3, retrochiasmatic **(K)**; and Type 4, intraventricular **(L)**.

Latter one being an important prognostic factor, different classifications have been proposed to integrate the exact tumor location, extent of preoperative hypothalamic involvement and postoperative damage in surgical planning and risk stratification. A classification by Puget et al. assessed the value of preoperative grading of the tumor according to the degree of hypothalamic involvement as follows: Grade 0, no hypothalamic involvement; Grade 1, the tumor abutting or displacing the hypothalamus; and Grade 2, hypothalamic involvement (the hypothalamus is no longer identifiable), followed by the analogous postoperative grading algorithm for hypothalamic damage. The authors demonstrated that its degree significantly correlated with patient outcome. Furthermore, they revealed that a thorough evaluation of the preoperative MR images was a helpful tool for stratifying patients and guiding the surgical strategy: GTR versus STR (12). Another classification was suggested using the mammillary bodies as location reference for the degree of hypothalamic involvement. Similarly, these authors concluded that the degree of hypothalamic involvement according to their anatomical classification has an impact on postoperative BMI and QoL and that the attempt of surgical tumor removal beyond the mammillary bodies increases the risk of morbid hypothalamic obesity (37). Flitsch et al. suggested an amendment of this classification by including the CP location, its relationship to the diaphragm sellae and the optic chiasm: Type 1 CP are located below the diaphragm sellae, whereas type 2 tumors are supradiaphragmatic and infrachiasmatic. Type 3 CP are located above the chiasm (usually retrochiasmatic, extending into the third ventricle). Type 3 should be subdivided into type A and B with respect to the mammillary bodies, since type B can be related to severe hypothalamic damage, when approached by aggressive surgery (38). A recent anatomical subclassification by Morisako et al. refers to the location of the tumor with respect to the diaphragm sellae and the optic chiasm. It divides CP into an intrasellar, prechiasmatic, retrochiasmatic, and intra-3^rd^ ventricular type, while taking the tumor origin into account (39). The above listed anatomical classifications and their variations are comprehensively illustrated in **Figure 1**.

**Figure 2:**
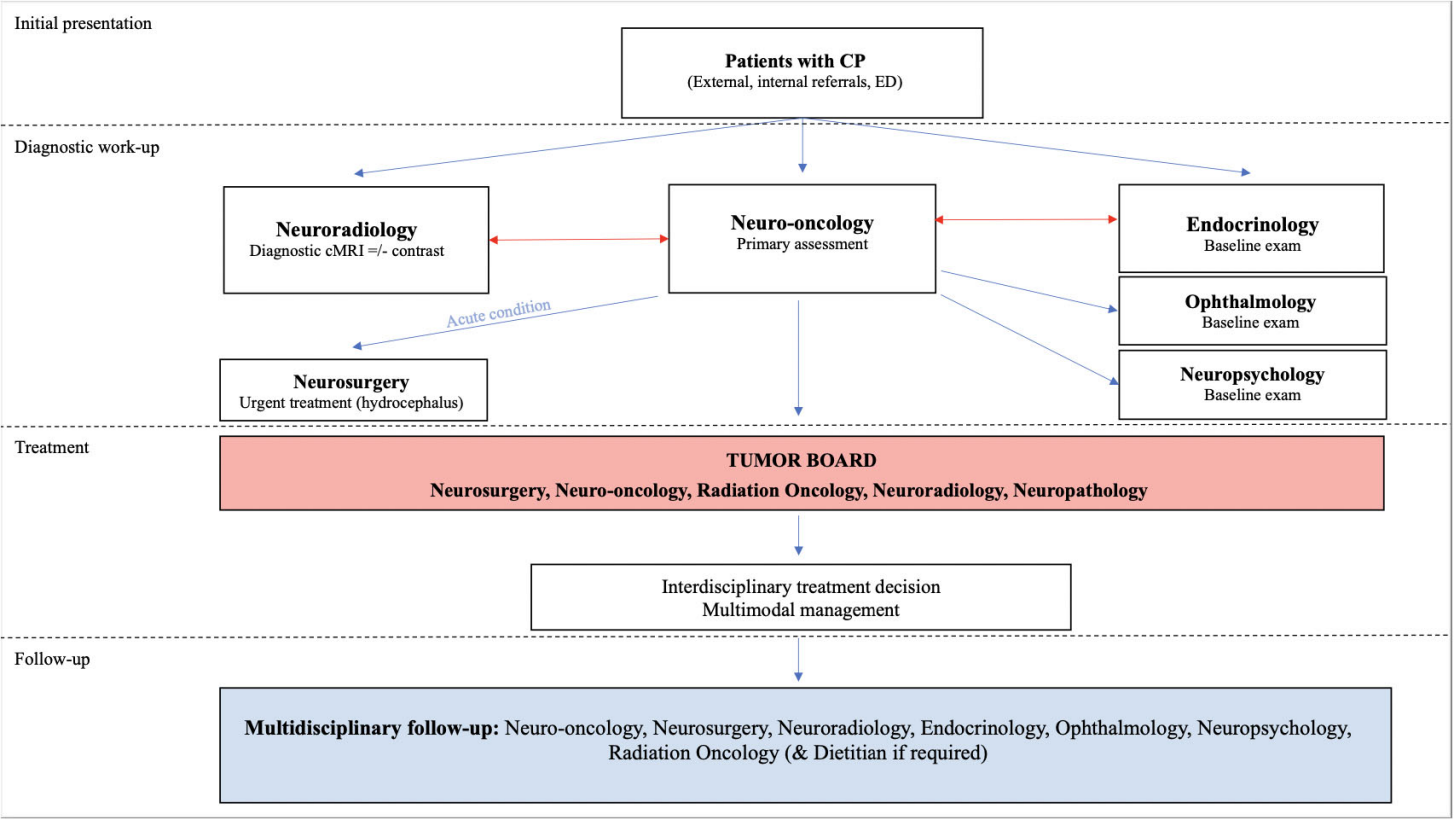
Flowchart illustrating the algorithm of diagnosis and management of CP including the aspect of multimodality and complexity of CP disease.

Further investigations in suspected CP patients, should include tests to generate baseline exams in all potentially involved specialties, such as ophthalmology, endocrinology, pediatrics, and neuropsychology. Additional diagnostic analysis may comprise histopathological and molecular genetic analysis once a biopsy or resection was performed and allowed to obtain tumor tissue. A schematic overview of involved disciplines and diagnostic pathways is given in **Figure 2**.

### Management

The benign nature of CP implied that complete resection was always considered as the ultimate cure and therefore the initial mainstay therapy in the 1990s. However, over the last decades an increasing number of studies have alternated this principle into a more variable and individual approach due to the awareness that GTR leads to unacceptable hypothalamic injury and that surgical invasiveness correlates with subsequent endocrinological dysfunction (11, 40). Currently, expanded imaging criteria are taken into account, which may influence the primary surgical strategy, such as exact tumor location and grading/classification in the MRI (as described above). Furthermore, precise assessment of involved functional structures may guide the choice of the right surgical approach: transcranial versus transsphenoidal, and the optimal extent of resection: STR versus GTR (19, 38). Other morphological criteria (cystic tumor components or the presence of hydrocephalus at the time point of diagnosis) may direct the surgeon towards other options like cyst decompression, Ommaya reservoir insertion (ORI), endoscopic procedures, or ventriculo-peritoneal (VP) shunt implantation (16, 41). Some surgeons also consider the presence of preoperative panhypopituitarism to justify a more aggressive approach, followed by their own capability of providing GTR in a safer manner.

Radiotherapy is mainly administered in CP residuals after STR or in tumor recurrences (34). Types of irradiation range from either conventional or fractionated conformal photon radiation to fractionated proton therapy. More uncommon approaches are hypofractionated frameless image-guided radiosurgery (CyberKnife radiosurgery, CKRS) or intracavitary colloid isotope application for cystic tumors (16). Irradiation side effects have been well described in the literature, and include endocrine, visual, and cognitive sequelae as well as vasculopathy and secondary malignancies (11, 42). Proton therapy provides a significant dose reduction and spares surrounding normal tissue suggesting a reduced side effect profile (43, 44). A comprehensive review on CP radiation techniques by Conti et al. concluded that CKRS holds the dose distributions and precision of frame-based techniques, such as single fraction gamma-knife radiosurgery, with the remarkable advantage of multiple-session treatments, which are better tolerated by sensitive peritumoral structures, such as the optic pathway and hypothalamus. Two studies investigated the application of CKRS in 54 CP patients, including a total of at least 4 children (exact number in one article not indicated) (45, 46). Their outcome results were superior with respect to complication rates, visual and endocrinological function compared those after single fraction gamma-knife radiosurgery. The authors concluded that CKRS may allow protection of visual and neuroendocrine function, especially for tumors located near the optic pathways and for large tumors (45). This, together with the comfort of a frameless technique, makes it an attractive option for the adjuvant post-operative treatment especially in children and young adults when GTR cannot be achieved, in those with hypothalamic involvement, and when the residual tumor is mostly solid (47). Yet, these options have a limited local availability and robust outcome data are still pending.

Additional treatment options are available for cystic CP, including cyst decompression or intracavitary installation of sclerosing substances such as interferon-alpha, *via* an intracystic catheter attached to an accessible Ommaya reservoir (48–52). A multicenter study reported that cyst shrinkage (>50%) was observed in 78% of treated patients after intracystic therapy with interferon-α (IFN-α), making it the most commonly applied intracystic agent (48). Bleomycin as a therapeutic agent for cystic CP became meanwhile obsolete due to its significant side effects, in particular neurotoxicity in case of leakage (53–56).

Secondary treatments in CP patients focus on alleviating chronic symptoms and on compensating endocrinological dysfunction caused by hypothalamic involvement/damage. They comprehend hormonal replacement as well as symptom-orientated care, but also measures of surveilling and ensuring quality of life (QoL) in these patients. The most frequent symptoms resulting from neuroendocrine dysfunction are obesity and eating disorders, followed by various co-effects, such as physical fatigue, social distancing and impacted psychosocial development (6). Obesity is observed in 12 to 19% of patients at the time of CP diagnosis, while another significant prevalence for severe obesity is seen in 55% CP patients within the first 6 to 12 months after surgery (6). Variable treatments of CP-induced obesity have been proposed and include increased physical activity, appetite regulation, pharmacological treatments as well as bariatric treatments with limited effectiveness (57). Ongoing trials aim to identify effective medications that show a higher response in obesity reduction. For example, a recently published randomized, placebo-controlled trial analyzed a once-weekly administration of 2.4-mg dose of subcutaneous semaglutide, in obese adolescents alone or in combination with a lifestyle intervention. They found that a combined approach resulted in a greater reduction in BMI than lifestyle intervention alone (58). Additional hormonal replacement therapies may include glucocorticoid, thyroid hormone, and sex hormone supplementation, medical treatment of diabetes insipidus as well as growth hormone replacement (59). While the latter is still a matter of controversy in some institutions, there is sufficient evidence to safely substitute growth hormone in the presence of growth hormone deficiency without increased risk for relapses (60, 61). Given the high complexity of CP patients with multiple symptoms or sequalae, the care pathway has to be individually adapted and follow a multidisciplinary approach - even on a long-term perspective, involving neurosurgeons, radiologists, radiation oncologists, endocrinologists, pediatric oncologists, and psychologists (15).

### Paradigm shift in treatment: function preservation

In the past, the majority of CP therapies focused on cure *via* radical tumor resection, followed by attempted medical compensation of hypothalamic dysfunction. However, the therapeutic goals for pediatric CP have changed and now rather focus on upfront function preservation. Accordingly, there has been a paradigm shift in management strategies of pediatric CP with growing worldwide advocacy for structure and function preserving techniques, for instance less aggressive surgical approaches (26, 40, 59, 62). In addition to changes in the extent of resection, the therapeutic approach to CP has evolved to include cyst decompression and intracystic chemotherapy *via* Ommaya reservoir insertion (ORI) (8, 63). ORI represents a minimally invasive intervention, which allows cystic decompression *via* aspiration and/or intracystic installation of agents, both designed to obtain durable cyst shrinkage with minimal overall toxicity (51, 52, 64). Given that pediatric CP are almost exclusively of the adamantinomatous subtype with frequent cyst formations (65, 66), instillation of intracystic agents is considered a valuable treatment option for recurrent monocystic CPs but also a primary treatment for large cysts with mass effect. Especially in very young patients with monocystic disease, intracystic therapies present a chance to avoid or at least to postpone aggressive surgery and radiotherapy to an older age (15, 52). Previous studies have addressed the efficacy of different agents, including bleomycin and IFN-α (56, 67–69). Especially, INF-α was found to delay disease progression and potentially offer a protracted time to definitive surgery or radiotherapy with a favorable toxicity profile compared with other therapeutic modalities (49, 70, 71). A recent study analyzed the impact of ORI on endocrine function in children with CP compared to surgical resection (72). Latter one lead in 62.5% of the patients to immediate post-operative endocrinological dysfunction, compared to 6.8% after ORI. Endocrine stability was maintained after ORI in 93.2% of the patients with a mean even-free survival (EFS) of 19.4 months (CI: 11.6-34.2). compared to 37.5% (odds ratio: 0.047 (CI: 0.004-0.263, p<0.0001) with a mean EFS of 13.4 months (CI:10.6-NA) after resection, hazard ratio: 0.460 (CI: 0.203-1.044, p=0.063) (72). Also, in patients with pre-existing deficits, it was observed that the number of dysfunctional endocrine axis remained stable after ORI, however, increased in patients, who underwent upfront resection. The ORI-related treatments in this study were variable and included intracystic administration of bleomycin or IFN-α, but also consisted of intermittent cyst fluid aspiration or no ORI-related treatment at all. The authors observed that a longer duration of anatomical decompression of the cyst (mass effect reduction) correlated with longer endocrinological function preservation and that if ORI resulted in a maintained decompression, intracystic therapy was unnecessary. Given that more than 30% of the analyzed patients did not receive any intracystic agent after ORI, and no difference in endocrine outcome in relation to the type of ORI-associated treatment was found, the assumption was made that already cyst drainage alone contributes significantly to preservation of endocrine function, analogous to the observation of Rachinger et al (73). These results indicate that function preservation can be successfully achieved by simple mass effect reduction while avoiding aggressive tumor resection. Recently opened trials using either MEK inhibitor (Binimetinib) or IL-6 inhibitor (Tociluzumab) may add to the potential armentarium of function preservation in children affected by CP.

### Role of multimodal management

The complexity and chronic nature of CP disease exceed the competences and capacities of single specialists and therefore warrants a multidisciplinary approach allowing to offer concise knowledge and variable treatment options for the individual needs related to each specialty. Accordingly, multimodal management of CP patients with complex conditions may involve multiple specialties at different time points or continuously, coordinated, and linked *via* regular interdisciplinary information exchange (**Figure 2**). Furthermore, the planning of follow up schedules including successful transition from pediatric to adult care providers will require a robust care network for health maintenance. Different setups and programs for CP patients are available at leading care institutions, however, coordination of multiple specialties in these excellence centers often remains a challenge (74). A similar complexity and high-intensity demand were observed in other diseases, such as in children with neurofibromatosis and lead to the establishment of centralized specialty clinics, providing comprehensive care while coordinating interdisciplinary follow ups and information exchange (75). Integration of such specialty clinics may under certain conditions and in specific centers contribute to further improvement of coordinated care, maintenance of follow up and care transition to adulthood.

## Discussion

Management of pediatric CP remains a challenge due to the complexity of the disease and persisting controversies regarding surgical treatment. Childhood-onset CP frequently manifests as a chronic disease and may go along with severe impairment of QoL repercussing from comorbidities caused by hypophysial, hypothalamic (panhypopituitarism, obesity, hyperphagia, obsessive food-seeking behavior, neuropsychological disorders), and neurological dysfunctions (12, 15). The state-of-the-art in the management of CP has recently been turning into multimodal strategies aiming to limit surgery- and radiation-related morbidity (21). The importance of adapting therapies to the goal of avoiding long-term sequalae, especially of endocrine deficits became more eminent and introduced the term “function preserving therapies”. Although some surgeons argue that in their hands GTR and cure can be achieved without any endocrinological sequelae, a generalized “function sparing” while being curative approach does not exist as such and entertains the dilemma of choosing the right therapeutic strategy. For instance, a multicenter prospective surveillance study (KRANIOPHARYNGEOM 2000) showed that high rate of early events in terms of recurrences after GTR (3y-EFS: 0.60 ± 0.10; n = 37) was observed during the first three years of follow-up (p = 0.007), underlining that even when GTR was achieved it does not automatically imply successful cure of disease (5). The major factor about function preservation in CP seems to be the anatomical mass effect reduction on hypothalamic structures, either *via* less aggressive surgeries or even more conservative therapies, for example ORI with cyst drainage.

Hence, the authors advocate for a multidisciplinary approach of CP patients involving all related experts. Although achieving treatment consensus within a multidisciplinary setting remains challenging, it offers a comprehensive and wide perspective on the individual patient’s needs. Another advantage of multimodal care is reciprocal knowledge exchanges about advances and new technologies in the field as available per each specialty. In this context better understanding of CP molecular biology recently resulted in development of targeted neoadjuvant treatments (BRAF/MEK inhibitors) for a subset of CP patients harboring the papillary subtype with a BRAFV600E mutations (76). Two studies reported a reduction in tumor volume > 85% after only 5 months while endocrine function remained stable (76, 77). These results are encouraging but require evaluation in larger studies and are not of significant relevance in the pediatric age group given that the papillary CP subtype is very rare in children. Other targeted therapies for ACP with CTNNB1 mutations and consecutive activation of the beta-Catenin dependent WNT-signaling pathway are underway (78). They will sooner than later play a major role in CP subtype-specific modulation and significantly contribute to the contemporary spectrum of function-sparing therapies (79).

## Conclusion

This narrative highlights the complexity of the natural history of pediatric CP, its requirement for multidisciplinary management and the importance of “function sparing” therapies. Treatment algorithms experienced several changes over the last decades and will be subject to constant optimization. Implementation of experienced interdisciplinary networks generating individual therapeutic strategies is mandatory to avoid or minimize long-term consequences for the patient. Alternative treatments, including intracystic ORI combined with other therapies or even molecular genetic approaches may evolve further and offer more robust opportunities for function preservation in CP patients.

## Data availability statement

The original contributions presented in the study are included in the article/supplementary material. Further inquiries can be directed to the corresponding author.

## Author contributions

L-NL performed the literature review and drafting of the manuscript. AP helped drafting the figures and reviewed the manuscript. UB and EK guided the manuscript design, supervised the quality of literature included and critically reviewed the manuscript. All authors contributed to the article and approved the submitted version.
